# Host Regulators of Liver Fibrosis During Human Schistosomiasis

**DOI:** 10.3389/fimmu.2018.02781

**Published:** 2018-11-28

**Authors:** Severin Donald Kamdem, Roger Moyou-Somo, Frank Brombacher, Justin Komguep Nono

**Affiliations:** ^1^Cape Town Component, International Centre for Genetic Engineering and Biotechnology, Cape Town, South Africa; ^2^Division of Immunology, Health Science Faculty, University of Cape Town, Cape Town, South Africa; ^3^Immunology of Infectious Diseases Unit, South African Medical Research Center, Cape Town, South Africa; ^4^The Medical Research Centre, Institute of Medical Research and Medicinal Plant Studies, Ministry of Scientific Research and Innovation, Yaoundé, Cameroon; ^5^Faculty of Health Sciences, Wellcome Centre for Infectious Diseases Research in Africa, Institute of Infectious Diseases and Molecular Medicine (IDM), University of Cape Town, Cape Town, South Africa

**Keywords:** liver fibrosis, host factors, human schistosomiasis, hepatosplenic schistosomiasis, wound healing

## Abstract

Liver fibrosis is a wound-healing process purposely aimed at restoring organ integrity after severe injury caused by autoimmune reactions, mechanical stress or infections. The uncontrolled solicitation of this process is pathogenic and a pathognomonic feature of diseases like hepatosplenic schistosomiasis where exacerbated liver fibrosis is centrally positioned among the drivers of the disease morbidity and mortality. Intriguingly, however, liver fibrosis occurs and progresses dissimilarly in schistosomiasis-diseased individuals with the same egg burden and biosocial features including age, duration of residence in the endemic site and gender. This suggests that parasite-independent and currently poorly defined host intrinsic factors might play a defining role in the regulation of liver fibrosis, the hallmark of morbidity, during schistosomiasis. In this review, we therefore provide a comprehensive overview of all known host candidate regulators of liver fibrosis reported in the context of human schistosomiasis.

## Introduction

Schistosomiasis represents the second most important parasitic disease in the world in terms of public health impact (1). With 120 million symptomatic (2) and 20 million with severe morbidity (3), schistosomiasis results in the loss of 70 million disability-adjusted life years (DALYs) (4) and preferentially occurs in developing countries with almost 200 million infected in Africa alone (5).

Schistosomes cause varying clinical symptoms and organ complications due to the species-specific tropisms of the egg-laying adult worms (6). While *Schistosoma haematobium* adult worms reside in vessels of the bladder causing urinary schistosomiasis, those of *Schistosoma mansoni* and *Schistosoma japonicum* reside in the mesenteric veins of the intestinal tract causing hepatosplenic schistosomiasis leading to progressive liver fibrosis and portal hypertension (6).

The immunology of schistosomiasis has progressed through the use of murine and non-human primate animal models, corroborating in many cases with human data (7, 8). The disease morbidity is driven by schistosome eggs and not directly by adult worms (9). Upon infection, a fraction of the produced eggs is not excreted by the host and become permanently lodged in organs such as the intestines and liver (*S. mansoni* and *S. japonicum)* or in the bladder and urogenital system (*S. haematobium)*. Highly immunogenic and cytolytic substances are released within the egg secretions (10). This induces a granulomatous and fibrotic response from the host largely characterized by T helper-2 cytokines (IL-4, IL-5, and IL-13), eosinophils and alternatively activated macrophage (11, 12). Host granulomas primarily form to contain and wall off the trapped toxic eggs products (13), which allows the host to live with the infection for many years. However, detrimental effects associated with persistent granulomas such as excessive collagen deposition leading to untoward fibrosis and portal hypertension arise, causing host pathology (13).

## Liver fibrosis during human schistosomiasis

The liver is an organ made by many specialized resident non-parenchymal cells, including Kupffer cells, liver sinusoidal endothelial cells and hepatic stellate cells (HSCs). First described by Kupffer in 1876 (14), HSCs which represent 5–8% of all liver cells (15) are located in the space of Disse in the liver sinusoid (16). These cells are responsible for maintenance of the extracellular matrix, storage of vitamin A (17), with a possible role in controlling blood flow through the liver (18). Upon stimulation, in response to injuries, normally quiescent HSCs (qHSCs) are activated HSCs (aHSCs), lose their ability to store vitamin A, increase expression of alpha-smooth muscle actin (α-SMA), a profibrotic gene, and develop a broader “stretched” cytoplasm. aHSCs then undergo a process of trans-differentiation toward collagen-producing liver myofibroblasts, the main cell type responsible for hepatic fibrosis (19).

Hepatic fibrosis comes from a variety of etiologies including hepatosplenic schistosomiasis (20), following the accumulation of parasitic eggs within the liver (21). Adult pairs of *S. mansoni* worms, particularly, reside within the mesenteric veins where females release on average 340 eggs per female per day with rates ranging between 190 and 658 eggs depending of the strain; this rate is higher for *S. japonicum* (22). More than 50% of eggs are carried to the liver by the portal circulation where they become trapped in the liver sinusoids (13). This is associated with the production of profibrotic cytokines (23, 24). Both murine and human infections with *S. japonicum* or *S. mansoni* reveal aHSCs as the key drivers of hepatic fibrosis (25, 26).

In the context of hepatosplenic schistosomiasis, an accumulating number of studies have reported disparities between prevalence of infection and levels of tissue morbidity, characterized by the stage of advancement of hepatic fibrosis and the presence/absence of periportal fibrosis (11, 27–30). For example, despite a higher prevalence and intensity of *S. mansoni* infections, in Kenya (31, 32) and Mali (33) compared to Egypt (31, 32) and Sudan (34, 35), the prevalence of periportal fibrosis in endemic sites is considerably higher in the latter countries. Furthermore, even adjacent communities with comparable levels of *S. mansoni* infection, exhibit considerable differences in their prevalence of periportal fibrosis (11). Although some possible explanations for these observed differential morbidity patterns could well be the different duration, intensity of infection (32, 35), the host genetic background (36, 37) has been increasingly suggested as a central basis for these discrepancies (11, 30, 38–42) as it appears that hepatic fibrosis occurs and progresses dissimilarly in schistosomiasis-diseased individuals with the same extrinsic and biosocial risk factors. In fact, in another recent study conducted in a village of rural Cameroon endemic for hepatosplenic schistosomiasis, participants with similar egg excretion profiles, similar age/gender distribution, same length of residence in the area, same frequency and duration of daily exposure to contaminated water, same social status and within similar dates of reinfection (i.e., previously treated with Praziquantel at the same time) as judged by the average number of eggs excreted in the feces, displayed a strikingly different stage of advancement of schistosomiasis-driven hepatic fibrosis (43). This indicated that, although the presence of the parasite eggs prompt the onset of liver lesions, host factors might indeed play a defining role in the regulation of liver fibrosis during hepatosplenic schistosomiasis. With the well documented and successful concept of host-directed therapies against diseases now including infectious diseases (37, 39, 42, 44, 45), the recollection of known host-derived factors that potentially play a role in the control of liver fibrosis during human schistosomiasis (pro- or anti-fibrotic) is long overdue for research on schistosomiasis in particular and all fibroproliferative diseases in general.

## Humans factors associated with liver fibrosis during schistosomiasis

**Figure 1:**
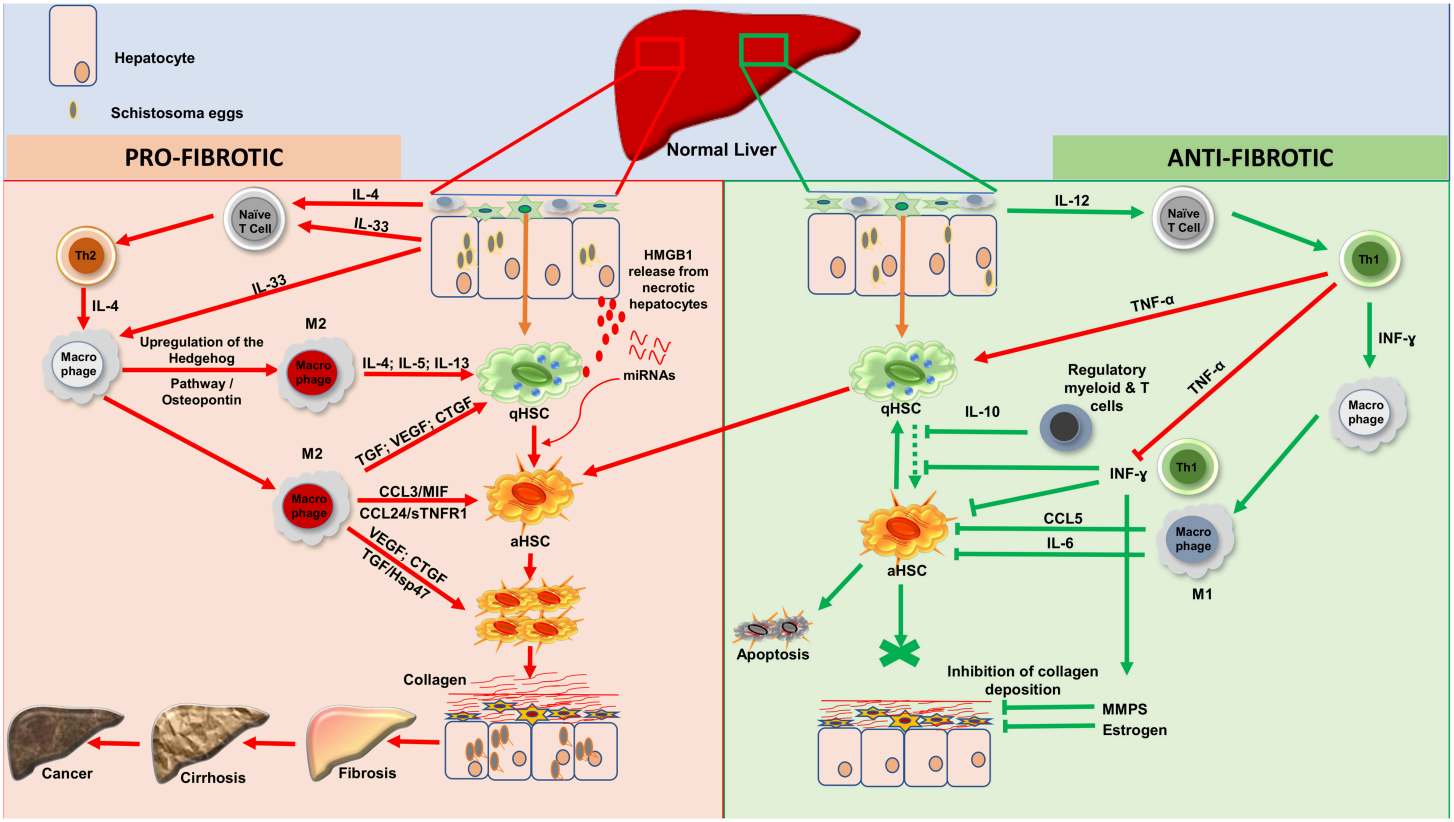
Processes of liver fibrosis progression and regression during human hepatosplenic schistosomiasis. **Profibrotic processes:** Upon stimulation, by *Schistosoma* spp. eggs antigens, injured hepatocytes will release IL-33 as a danger alerting molecule. Moreover, schistosome eggs will drive, via cells such as Innate lymphoid cells, the release of IL-4. IL-33 and IL-4 will act on naïve T cells to promote Th2 differentiation. IL-33 will also induce through his receptor ST2 located on macrophages, the upregulation of the Hedgehog pathway which will then drive via osteopontin the alternative activation of macrophages (M2). The effect of IL-33 on endothelial cells will also promote angiogenesis which will increase the vascular permeability. With the support of TNF-α produced by Th1, Th2 cells will then produce large amount of type 2 cytokines (IL-4, IL-5, and IL-13) which will induce the activation of qHSCs. M2 differentiated under the flow of Th2-released type-2 cytokines can also foster the activation of qHSCs by releasing TGF- β1, VEGF or CTGF. While the two latter can directly stimulate the proliferation of aHSCs, TGF-β1 can act through the Hsp47. This action is sustaining by some chemokines especially (CCL3; MIF; CCL24 and sTNFR1). Moreover, necrotic hepatocytes might release High mobility group box 1 (HMGB1) protein as an alarmin that will ultimately foster HSC activation. In the midst of advanced fibrosis, human livers might further release miRNAs that alter host metabolism, triggering HSC activation to promote tissue fibrosis. Altogether, proliferating aHSCs will produce a very large amount of collagen, which will accumulate and lead to fibrosis which can progress to cirrhosis and then ultimately to liver cancer. **Anti-fibrotic processes:** Three possibilities can account for regression of fibrosis: apoptosis; senescence or reversion of aHSCs to their quiescent stage. Upon stimulation, hepatic resident macrophages will produce IL- 12 which will drive the differentiation of Naïve T-cell to Th1. Th1 cells will release INF-ɤ which will classically activate macrophages (M1). Various regulatory T and/or myeloid cells are also known to produce IL-10 which can block the activation of qHSCs to consolidate an anti-fibrotic effect. This action is further supported by the large amount of INF-ɤ produced by Th1 although balanced by the level of secreted TNF-α. aHSCs might return to the quiescent/senescent state under the action of IL-6; CCL5 or INF-ɤ or undergo apoptosis depending on how strong the profibrotic signal was. INF-ɤ can also activate MMPs to digest the deposited collagen and this action is reinforced by estrogen in women. IL, Interleukin; HSCs, Hepatic Stellate Cells; qHSCs, quiescent Hepatic Stellate Cells; aHSCs, activated Hepatic Stellate Cells; TGF-β1, Transforming Growth Factor beta 1; VEGF, Vascular Endothelial Growth Factor; CTGF, Connective Tissue Growth Factor; MIF, Migration Inhibitory Factor; INF-ɤ, Interferon gamma; TNF-α, Tumor Necrosis Factor alph; MMPS, Matrix Metalloproteinase; sTNFR1, Soluble Tumor Necrosis Factor Receptor-1; CCL3, Chemokine C-C motif ligand 3; CCL5, Chemokine C-C motif ligand 5; CCL24, Chemokine C-C motif ligand 24; M1, Classical activated Macrophages; M2, Alternatively activated Macrophages; Hsp47, Heat Shock Protein 47; Th1, Type 1 Lymphocytes; Th2, Type 2 Lymphocytes. HMGB1, High mobility group box 1. NB, These 2 cascades of processes take place simultaneously during the host attempt to heal the wound after injury by trapped schistosome eggs.

The present compilation of host factors reported to associate with the progression/resistance to hepatic fibrosis during Human hepatosplenic schistosomiasis (Table 1 and Figure 1) should facilitate subsequent investigations aimed at validating their role as hepatic fibrosis monitoring tools and for the design of host-directed intervention strategies against morbidity due to hepatic fibrosis during schistosomiasis.

**Table 1:** Reported Pro and anti-fibrotic human host factors during hepatosplenic schistosomiasis.

**PROFIBROTIC vs. ANTI-FIBROTIC**	
**Profibrotic factors**	**References**
Type 2 Cytokines (IL-4; IL-5 and IL-13)	(41, 42, 46–51)
Interleukin 33 Receptor (ST2)	(52)
Tumor Necrosis Factor alpha (TNF-α)	(11, 30, 44, 49, 53–55)
CCL3	(56)
CCL24/sTNFR1/MIF	(57)
Connective Tissue Growth Factor (CTGF)	(39, 58–60)
Transforming Growth Factor-beta 1 (TGF-β1)	(61)
Vascular Endothelial Growth Factors (VEGF)	(62, 63)
Hedgehog ligand (Hh)	(64)
Osteopontin (OPN)	(65–67)
Antibodies (IgG4 and IgE)	(68–71)
Eosinophils/Eosinophil cationic protein (ECP)	(49, 72)
Mannose-Binding Lectin (MBL)	(73)
MicroRNAs	(74)
High mobility group box 1 (HMGB1)	(75)
**Anti-fibrotic factors**	**References**
Interferon gamma (INF-ɤ)	(8, 11, 30, 36, 37, 40)
Interleukin 6 (IL-6)	(51)
Interleukin 10 (IL-10)	(11, 40, 45, 51)
Chemokines**:** RANTES (CCL5)	(11)
Major Histocompatibility Class II (MHC II)	(76–78)
Regulatory T cells (Tregs)	(79)

### Cytokines and cytokine receptors

Abundance of information from murine studies links type 2 cytokines (Th2) rather than Type 1 (Th1) responses with the development of hepatic fibrosis (80, 81). These studies have defined a dominant role for Th2 immune mediators in the liver fibrotic process during hepatosplenic schistosomiasis (80, 82). As a telling example, a recent finding in mechanistic chronic murine schistosomiasis model, has demonstrated an amelioration of liver fibroproliferative pathology following the transgenic reduction of IL-4 receptor alpha-mediated signaling (81). A parallel is now emerging in human studies. High levels of IL-4 and IL-13 (secreted primarily by type-2 immune cells such as Th2 cells to act toward the alternatively activation of macrophages into M2 and the activation of Hepatic stellate cells, Figure 1) were found to associate with periportal fibrosis progression during schistosomiasis in infected individuals from Brazil, Bahia, Philippines and Zambia (42, 46, 47, 49, 51). In addition, an IL-13 promoter enhancing single nucleotide polymorphism (SNP), rs1800925, has been shown to strongly associate with a higher risk of pathological hepatic fibrosis in *S. japonicum*-infected individuals (50).

Another type-2 cytokine receptor, ST2 in its soluble form (sST2), has been reported to be present at high level in the serum of diseased patients with advanced hepatic fibrosis during *S. japonicum* infection (52).

In sharp contrast, the canonical Th1 cytokine INF-ɤ has been associated with fibrosis reduction during human schistosomiasis. In Sudanese patients, higher levels of INF-ɤ were associated with a marked reduction of the risk of fibrosis during schistosomiasis (11, 30). Additionally, lower levels of INF-ɤ were linked to a higher risk (more than 8-fold comparing to people with large amount of INF-ɤ) of periportal fibrosis in patients from China and Uganda (11, 40). Further investigations at a molecular level revealed that a polymorphism in the INF-ɤ-receptor 1 (IFNGR1) gene which lead to reduced functionality of the receptor, may account for increased susceptibility to severe fibrosis (8). In addition, INF-ɤ gene SNP, translating into reduced gene transcription, associated with more severe fibrosis in Sudanese patients (8, 36, 37) supporting a protective action of INF-ɤ against hepatic fibrosis during schistosomiasis. These reports are consistent with the powerful antifibrogenic role of INF-ɤ (primary product of type-1 NK and Th1 cells and acting predominantly on macrophages to drive M1 differentiation) which inhibit the transdifferentiation of HSCs into myofibroblast, reduces the production of extracellular matrix proteins and increases the collagenase activity of the liver (83). Finally, IL-6 (secreted by pro-inflammatory cellular responses such as that of classically activated macrophages M1 to counter HSC activation) was also claimed to be protective against severe fibrosis by the team of Mutengo (51) who demonstrated that IL-6 was expressed considerably more by *S. mansoni* egg-stimulated whole blood cultures from individuals with no or mild fibrosis when compared to cultures of individuals with moderate/severe fibrosis (51). This is consistent with reports that associate the lack of IL-6 with increase in liver injury (84) and the recent potential therapeutic ability of IL-6 to reverse liver fibrosis (85).

Intriguingly, as another Th1-related cytokine, Tumor necrosis alpha (TNF-α) at higher levels in supernatants from patient's whole-blood cultures, rather strongly associated with an increased risk of periportal fibrosis during hepatosplenic schistosomiasis (11, 30, 49, 53). In fact, the TNF-α 308.2 SNP or rs1800629(AA), translating into an increased transcription of the TNF-α gene (55), has been shown to correlate with a more severe hepatic fibrosis during *S. mansoni* infections (54). The picture is a clearly more nuanced as a study in Uganda observed a differential association between INF-ɤ, TNF-α and fibrosis (86) where TNF-α reportedly acted by balancing the protective effect of INF-ɤ (87); a trend confirmed in Zambia (51). Overall, adding to observations in experimental models, a strong case is made for INF-ɤ as an anti-fibrotic cytokine (11, 30), whereas TNF-α (also produced by Th1 cells and macrophages) primarily appears to aggravate hepatic fibrosis during schistosomiasis (87).

Reports on the possible role of IL-10 (secreted by regulatory cells such as regulatory myeloid and/or T cells) in relation to hepatic fibrosis made mention of a regulatory role of the *il-10* gene SNPs on the progression of periportal fibrosis (45). In fact, higher risk of severe periportal fibrosis was reported when IL-10 production by blood mononuclear cells from schistosomiasis-diseased patients was low (11, 40, 51) suggesting a protective role of IL-10 against hepatic fibrosis. The anti-inflammatory and immunosuppressive potential of IL-10 which regulate the inflammatory process, central to tissue fibrosis, could be an explanation for these observations (88).

Consequently, the interplay between the arms of the immune response, as defined by the host overall cytokine profile, controls the inflammatory response and determines the evolution of liver fibrosis during schistosomiasis.

### Chemokines

In the last decades, many studies have shown that tissue injury leads to the production of chemokines that orchestrate cell trafficking to the site of injury during infectious diseases (89). In fact, peripheral blood mononuclear cells from schistosome-infected patients with ultrasonographically-defined hepatosplenomegaly produced high levels of CCL3 when exposed to egg antigen; and marked them as group under greater risk of developing severe disease with hepatic fibrosis (90). This observation was corroborated by independent data obtained from murine models which reported a less severe granulomatous response in CCL3-deficient mice following schistosome infection (56, 90). The findings were further confirmed in another study where the plasma concentrations of CCL3 and that of other chemokines, namely CCL24, sTNFR1 and MIF (migration inhibitory Factor), were associated with hepatic fibrosis progression (57).

RANTES (CCL5), another monocyte/macrophage-derived chemokine which has a central role in the regulation of both Th1 and Th2 responses by controlling the migration and activation of leukocytes on the site of inflammation (91), was found to negatively associate with periportal fibrosis (11).

Overall, these observations are consistent with published reports that showed that chemokines affect several processes, including differentiation and activation of fibroregulatory cells including fibroblasts which are central to the onset and progression of tissue fibrosis (56).

### Growth factors

Many Growth factors are known to be fibrogenic and thus contribute to hepatic fibrosis in schistosome infections independently or in association with other factors (23). Dessein et al. in a primary study reported that several SNPs (rs9402373, rs12526196, rs9402373, and rs1256196) that lie close to the Connective Tissue Growth Factor (CTGF) gene are associated with severe hepatic fibrosis in individuals affected by hepatosplenic schistosomiasis (39, 60). This observation gains support from the observation that CTGF overproduction, by cells such as M2 macrophages, was proposed to drive tissue fibrosis (61). In fact, CTGF has now been shown to promote fibroblast proliferation, migration, adhesion, and extracellular matrix formation (58, 59). Notably, CTGF is mostly upregulated by TGF-β1 (23), a well-known fibrogenic growth factor (also secreted by M2 macrophages) which was also identified to be positively associated through the heat shock protein 47 (Hsp47) with hepatic fibrosis in Chinese patients infected with *S. japonicum* (61).

Apparent controversial results were found for the implication of Vascular Endothelial Growth Factor (VEGF) in periportal fibrosis. Egyptian patients with periportal fibrosis had significantly elevated serum levels of VEGF (again secreted by cells such as M2 macrophages) when compared to non-diseased controls (62) whereas no significance difference in serum levels of this factor was reported in Brazilian cohorts of hepatosplenic schistosomiasis patients with or without hepatic fibrosis (63). This difference could be attributed to the small sample size used in the latter study (28 participants vs. 90 in the Egyptian study) and the heterogeneity in the case and control groups. Conclusively, a case is maintained for the clinical evidence that circulating levels of VEGF correlate with hepatic fibrosis progression considering its known role as regulator of angiogenesis, a crucial step in the process of fibrosis (62).

### Hedgehog ligands/osteopontin

Macrophages when activated via the alternative pathway, are instrumental in the liver fibrosis process (92). Understandably, Hedgehog (Hh) ligands, produced by macrophages were found to promote hepatic fibrosis during schistosomiasis by a reverse feedback action leading to an alternative activation of macrophages (64). This is consistent with the now defined role of the Hedgehog pathway in the onset and progression of vascular remodeling, regulation of HSC activation thus schistosomiasis-related fibrosis (64). In fact, Syn et al. elegantly demonstrated that the pro-fibrogenic effects induced by the Hedgehog pathway are mediated by a secreted host phosphoprotein, osteopontin (93). This is substantiated by the observation that high osteopontin level in serum and liver tissue correlates with hepatic fibrosis during schistosome infection (65, 66). This correlation was further validated in mechanistic chronic murine schistosomiasis model where higher levels of osteopontin release by macrophages associated with hepatosplenic disease (65, 66). Fundamentally, these findings are consistent with the demonstrated role of osteopontin in inflammation, immunity, angiogenesis, fibrogenesis and carcinogenesis in various tissues (94).

### Antibodies (IgG4 and IgE)

The isoform 4 of the Immunoglobulin class G (IgG4) was found 20 times elevated in patients with chronic schistosomiasis compared to non-infected controls (71). A role for IgG4 in schistosome-driven liver fibrosis was more elegantly defined as association was demonstrated between the former and liver fibrosis following schistosome infection (68–70). These observations were extended to Immunoglobulin E (IgE) which was also associated, although to a lower extent, to disease severity (69, 70). Together, these studies have defined, pending in-depth empirical studies, a possible role for host Immunoglobulins, of the G4 and E types, in the fibrotic process resulting from human schistosome infections (70).

### Eosinophil markers and cation protein

Other immune cells do also participate by their products in the process of hepatic fibrosis during hepatosplenic schistosomiasis. Eosinophils which play an important protective role in the immune response to *S. mansoni* infection (95), upregulate activation-related surface markers (CD69 and CD23) in response to host-released Type 2 cytokines, which altogether selectively associate with periportal fibrosis progression during hepatosplenic schistosomiasis. Moreover, Eosinophil Cationic Protein (ECP), a protein secreted by Eosinophils that efficiently kills *S. mansoni* at the larval stage, also affect fibroblast function. Eriksson et al. (72) identified the ECP 434GG genotype, which translate into a higher cytotoxic potential (96) as being associated with periportal fibrosis during hepatosplenic schistosomiasis (72). These observations are consistent with the suggested role of eosinophils in wound-healing, remodeling, development of post inflammatory fibrosis (72, 97) and the association of tissue eosinophilia and eosinophil degranulation with several other fibrotic syndromes (98). Moreover, human eosinophils express all isoforms of the Transforming Growth Factor beta (TGF-β1,2,3) (99) hinting to a possible role in the liver fibrosis process during human hepatosplenic schistosomiasis.

### Mannose-binding lectin (MBL)

In the northeastern of Brazil, Mannose-Binding Lectin which also acts as an opsonin released by mononuclear innate cells (monocytes, macrophages, dendritic cells), was reported to be a risk factor for advanced periportal fibrosis in chronic hepatic diseases as well as *S. mansoni* infection (73). This can be explained by the role of MBL as a central component of the innate immune response and its highly collagenous activity following host infection by invading pathogens (100).

### HLA class II

Positive and negative associations between HLA class II alleles and the risk of individuals developing moderate to severe hepatic fibrosis following *S. japonicum* infection were reported. While alleles HLA-DRB1^*^1501; HLA-DQB1^*^0601; DRB1^*^11011, DRB1^*^0409, and DRB1^*^0701 were associated with resistance to hepatic fibrosis; some other HLA-DRB1 alleles (HLA-DRB1^*^0901; HLA-DRB1^*^1302; DRB1^*^1202, DRB1^*^1404, and DRB1^*^1405) and Two HLA-DQB1 alleles (HLA-DQB1^*^0303 and HLA-DQB1^*^0609) were in contrast significantly associated with rapid progression of hepatic fibrosis (77, 78). In addition, HLA-DPA1^*^0103 and DPB1^*^0201 haplotypes were also associated with protection from both moderate and severe fibrosis (76).

### MicroRNAs

Some microRNAs were reported for the first time through miRNome and transcriptome analyses of livers from humans infected with *Schistosoma japonicum* in China, to play an important role in schistosomiasis-driven hepatic fibrosis. Notably, hsa-miR-150-5p, hsa-miR-10a-5p, hsa-miR-199a-3p, hsa-miR-4521, hsa-miR-222/221, hsa-miR-663b, and hsa-miR-143-3p were found to be upregulated in schistosome-infested fibrotic livers (74). These observations could be explained by the defined role of some miRNAs (hsa-miR-222/221 and hsa-miR-10a), respectively, reported to drive the activation of HSCs (101), or to upregulate the expression of TGF-β, a profibrotic growth factor (102); thus corroborating accumulating studies which report predominantly on an upregulation of fibrogenic microRNAs during schistosomiasis (103, 104).

### Regulatory T cells

Very few studies have focused on the role of regulatory T cells (Treg cells) on liver fibrosis during schistosomiasis. Notwithstanding, in the Hunan province China, high blood Tregs level was associated with severe hepatic fibrosis caused by *Schistosoma japonicum* (79). This observation aligned with another study which linked a high blood Tregs level with *S. mansoni* infection (105). A possible explanation for these observations is the clear anti-inflammatory thus anti-fibrotic role of Tregs that accumulate in the blood but failed to be recruited to the liver site of fibrogranulomatous inflammation during schistosomiasis. In fact, a decreased expression of tissue homing markers was reported on Tregs found in the blood of patients with severe fibrosis during schistosomiasis (79). Consequently, a deficit of recruitment of Treg cells to the fibrotic tissue, but rather the accumulation of these Treg cells in the blood, would explain the aggravated tissue fibrotic profile that coincides with elevated blood Tregs during schistosomiasis-driven severe liver fibrosis. An anti-fibrotic role for the host Treg cells is therefore suggested during human hepatosplenic schistosomiasis.

### High mobility group box 1 (HMGB1)

Originally described as a nuclear protein (106), emerging studies have showed that HMGB1 is closely associated with fibrotic disorders, including liver fibrosis (107). In this regard, a study conducted in Brazil reported high levels of HMGB1 in the plasma of both acute and chronically infected-patients with *S. mansoni* when compared with the sera of healthy donors (75). This was further supported in a study highlighting a significantly increased level of HMGB1 in patients with liver fibrosis caused by hepatitis B (108). Additionally, a former study reported that HMGB1 up-regulates alpha-smooth muscle actin (α-SMA) expression in HSCs and then drive fibrosis (109). This trend gained further strength in animal-model-based study where a HMGB1 inhibitor was able to downregulate the production of the profibrotic cytokines IL4, IL-5, and IL-13 and significantly reverse (over 50%) liver fibrosis (75).

## Concluding remarks

In conclusion, hepatic fibrosis is a very complex process that involves biosocial, genetic, cellular and soluble mediators. Most of these host-derived actors generally come into play as a response to the infection in order to protect the host but progressively drive the substance of the hepatosplenic schistosomiasis morbidity when overly solicited. It is now clear from experimental models and human studies that the expression or lack of expression of such host factors centrally regulate this process. While the factors reported in the present review were identified as independent players (Table 1), the regulation of hepatic fibrosis progression during hepatosplenic schistosomiasis is most likely multifactorial (Figure 1) and will necessitate more studies incorporating this concept. With the alarmingly limited efficiency of mass deworming campaigns alone to eliminate the disease and control the morbidity in resource-limited areas (43), a comprehensive assessment of these host factors is desperately needed as they constitute an attractive avenue for novel disease control strategies. To be used as drug targets, reported human host factors should be validated in animal models to evaluate their potential for therapeutic development. In fact, the targeting of identified profibrotic cytokines (IL-5; IL-13) and growth factors (CTGF; TGF-β1 and VEGF) should be pursued in conjunction or as an alternative to current disease control measures to attempt to alleviate the fibrosis process during hepatosplenic schistosomiasis. Other attractive candidate targets such as miRNAs should also be targeted, with the hope of reducing HSC proliferation, contractility and suppress angiogenesis by indirect mitigation of the TGF- β and VEGF-mediated signaling nodes. Encouragingly, this targeting approach is now being undertaken for some suspected profibrotic factors with promising results (75, 81). Complementarily, given the advancement of high-throughput analysis tools such as next-generation sequencing, the comprehensive identification and clinical validation of host factors that might regulate hepatic fibrosis individually and/or collectively in some schistosomiasis-diseased individuals should be further pursued to provide more candidates for therapeutic evaluation.

## Author contributions

JN and SK defined the topic and designed the search strategy. SK gathered and read articles, as well as wrote the first draft of the manuscript under the supervision of JN. JN corrected and validated the manuscript. JN, SK, FB, and RM-S did a thorough revision of the manuscript. All authors read and approved the final manuscript.

### Conflict of interest statement

The authors declare that the research was conducted in the absence of any commercial or financial relationships that could be construed as a potential conflict of interest.
